# Risk Factors for Alveolar Echinococcosis in Humans

**DOI:** 10.3201/eid1012.030773

**Published:** 2004-12

**Authors:** Petra Kern, Andrea Ammon, Martina Kron, Gabriele Sinn, Silvia Sander, Lyle R. Petersen, Wilhelm Gaus, Peter Kern

**Affiliations:** * University of Ulm, Ulm, Germany;; † Robert Koch-Institut, Berlin, Germany;; ‡ Health Authorities (Gesundheitsamt) of Charlottenburg-Wilmersdorf, Berlin, Germany;; § Centers for Disease Control and Prevention, Fort Collins, Colorado; USA

**Keywords:** alveolar echinococcosis, risk factors, case-control study, research

## Abstract

We conducted a case-control study to investigate risk factors for acquiring autochthonous alveolar echinococcosis in Germany. Forty cases and 120 controls matched by age and residence were interviewed. Patients were more likely than controls to have owned dogs that killed game (odds ratio [OR] = 18.0), lived in a farmhouse (OR = 6.4), owned dogs that roamed outdoors unattended (OR = 6.1), collected wood (OR = 4.7), been farmers (OR = 4.7), chewed grass (OR = 4.4), lived in a dwelling close to fields (OR = 3.0), gone into forests for vocational reasons (OR = 2.8), grown leaf or root vegetables (OR = 2.5), owned cats that roamed outdoors unattended (OR = 2.3), and eaten unwashed strawberries (OR = 2.2). Sixty-five percent of cases were attributable to farming. Measures that prevent accidental swallowing of possibly contaminated material during farming or adequate deworming of pet animals might reduce the risk for alveolar echinococcosis.

## Abstract 2

A case-control study of alveolar echinococcosis cases in Germany identifies several risk factors for the disease.

Human alveolar echinococcosis is caused by the larval stage (metacestode) of the fox tapeworm *Echinococcus multilocularis*, which usually develops in the liver of infected persons. Slow larval growth results in an asymptomatic phase of several years before diagnosis. When left untreated, the condition is lethal (*1*). Although modern treatments have considerably improved survival, complete cure is rare (*2**–**4*).

*E. multilocularis* occurs worldwide in many arctic and temperate zones of the northern hemisphere (*5*). Its life cycle is predominantly sylvatic; several carnivorous species such as the fox, wolf, and coyote serve as definitive hosts that excrete the eggs in their feces. Several small rodent species, such as the vole, lemming, and muskrat, serve as intermediate hosts; they become infected by oral intake of the eggs, and the larvae develop in their liver. In Europe, the red fox (*Vulpes vulpes*) is the main definitive host. In Germany, the parasite is endemic in many regions; the prevalence in red fox populations is <1%–>60% (*6*). Dogs and cats can also become infected as definitive hosts, but their infection rates are low (*7*).

Human infections follow accidental ingestion of infective eggs. From 1982 to 2000, a total of 126 alveolar echinococcosis patients with autochthonous infections reportedly received treatment in German clinics (*8*). In spite of these low case numbers, the disease is an important public health problem because of the high frequency of infections in specific geographic clusters (*8*), the severity of organ damage in cases of infiltrative parasitic growth or hematogenous spread, and the necessity for costly long-term treatment and follow-up (*3*). Current hypotheses of possible routes of transmission of the eggs to humans include infection by hands contaminated from the fur of infected animals (foxes, dogs, or cats) or from soil while gardening or during field work; eating contaminated uncooked food from fields or gardens; drinking contaminated spring water; or inhaling dust containing tapeworm eggs, possibly during field work. Only three published case-control studies have assessed risk factors for human infections. In Alaska, dog ownership, living in houses directly built on the tundra, and keeping dogs tethered near the house were identified as important risks (*9*). In Austria, cat ownership and hunting were associated with alveolar echinococcosis, while farming and dog ownership were not (*10*). In Japan, persons with a clinical diagnosis of alveolar echinococcosis or a positive serologic result were more likely than controls to have reared cattle or pigs or to have used well water (*11*). We investigated possible risk factors for acquiring human alveolar echinococcosis in Germany.

## Methods

We conducted a matched case-control study. Cases were selected from the European Echinococcosis Registry at the University of Ulm (*8*). An eligible case-patient was defined as a person 1) with positive histopathology of alveolar echinococcosis, or with positive morphologic findings by imaging techniques (ultrasound, computer tomography, magnetic resonance imaging) compatible with alveolar echinococcosis with or without serologic findings for the disease, 2) who was first diagnosed from 1990 to 2000, and 3) who lived in Germany and was still alive. The time frame of diagnosis was restricted to reduce possible recall bias, and only live patients were included to avoid possible information bias introduced by interviewing the relatives of cases. Fifty-three patients were eligible; of these, 40 participated in the study, 11 refused participation, and 2 gave consent after the study was completed.

Controls were individually matched to case-patients by age and place of residence. Matched residences were those in which the patients had lived during the 10 years before diagnosis. If the patients had moved during this time, the residence where they had lived for the longest period was chosen. Potential controls were contacted by random-digit telephone dialing. For every residence (5-digit postal code), 100 randomly selected numbers from the electronic version of the telephone directory were provided by ZUMA (Centre for Survey Research and Methodology, Mannheim, Germany). Eligible controls were persons who had lived in the municipality during the same period as the patients for at least 1 year, and who were of the same age (±5 years). Three controls for each of the 40 cases were chosen to detect an odds ratio of 3.0, assuming a frequency of a single exposure of 20% among controls and 43% among patients, with a power of 80% and a two-sided significance level of 5%.

Exposure information was obtained with a standardized questionnaire administered by telephone from February to August 2000. Specific behavior and activities during the 10 years preceding the diagnosis of an individual case were assessed. Persons were considered to be dog or cat owners or to have farmed if the duration exceeded 1 year. Deworming of dogs and cats was rated as an effective prophylactic anthelmintic measure only when performed at monthly intervals. For dwellings, gardens, and meadows, a close vicinity to possibly contaminated areas was defined as being <100 m from meadows, forests, fields, or rivers. Eligible patients were asked for their written informed consent; controls were asked for their oral informed consent before the interview. All data were processed without personal identifiers. The ethical committee of the University of Ulm approved the study protocol.

Statistical data were analyzed with SAS Version 8.2 (SAS Institute Inc., Cary, NC). For variables that might influence the occurrence of alveolar echinococcosis, the crude odds ratio (OR), the 95% confidence interval (CI), and the p value were calculated from simple conditional logistic regression (*12*). For each risk factor with a p value <0.05, the attributable risk was calculated by multiplying the proportion of exposed among the cases by (OR–1)/OR. All exposure factors strongly associated with the disease (p values <0.05) and independent of each other (Cramers's V <0.5) were combined in a specific risk score. Of factors with high interdependencies, only one was chosen according to its contextual relevance as compared to the other variables. The score was computed for each participant by adding 1 point for each specific exposure when this factor was present. The distribution of the score points in cases and controls was described by a boxplot. A stratified analysis for farmers and nonfarmers was performed; the regression models, including interaction terms of farming with the 10 other exposure factors of the risk score, showed that the effects of none of the factors were clearly distinct between the two groups (results not shown).

## Results

Forty cases and 120 controls took part in the study. The gender distribution differed between patients and controls. Only 22% of the patients were <50 years of age, 73% were 50–79, and 5% were >79 (range 15–82 years). The educational status was similar among patients and controls. Most study participants lived in small villages (Table), and most in southern Germany, only 10% lived in central and northern Germany. Of the patients, 36 had lived in these places for >20 years; 4 had moved during the possible exposure time.

**Table:** Characteristics of the study population

Demographic characteristics	Patients N = 40	Controls N = 120
	n (%)	n (%)
Age (in y) at time of interview^a^		
<20	1 (2)	
20–29	2 (5)	
30–39	4 (10)	
40–49	2 (5)	
50–59	10 (25)	
60–69	10 (25)	
70–79	9 (23)	
>79	2 (5)	
Sex		
Male	18 (45)	41 (34)
Female	22 (55)	78 (65)
Data not available	– –	1 (1)
Education		
Secondary school	28 (70)	74 (62)
Intermediate level	7 (18)	29 (24)
Grammar school	4 (10)	14 (11)
Left school early	0	1 (1)
Still in school	1 (2)	1 (1)
Data not available	0 -	1 (1)
Completed vocational training		
Yes	27 (68)	87 (73)
No	13 (32)	30 (25)
Data not available		3 (2)
Population of hometown^a^		
<200	4 (10)	
200–<600	10 (25)	
600–<7,000	21 (53)	
7,000–<20,000	2 (5)	
>20,000	3 (7)	
House at town outskirts		
Yes	26 (65)	85 (71)
No	14 (35)	34 (28)
Data not available		1 (1)
Duration of residence at assumed place of exposure		
>30 y	31 (78)	
20–29 y	5 (13)	
10–19 y	3 (7)	
<10 y	1 (2)	

^a^Controls were matched to patients by age and hometown.

Simple conditional logistic regression analyses indicated 22 possible risk factors that were more common among patients than controls (p values <0.05) (Table A1). Patients were more likely than controls to have owned dogs (OR = 4.2), and several characteristics, such as leaving the dog in the garden unattended (OR = 6.1) or killing game (OR = 18.0), were more common among dogs belonging to patients (Table A1). Patients were also more likely to have dewormed their dogs at infrequent intervals (OR = 5.6). Six persons in the study population reported hunting; one patient and two controls had hunted foxes, all for long periods (18–45 years). Owning cats that roamed outdoors unattended (OR = 2.3) and cats that ate mice (OR = 2.3) were more common factors for patients than controls.

Patients were more likely to be farmers (OR = 4.7); attributable risk calculations suggested that farming could account for almost two thirds of the infections. Specific farming activities were more common among patients than controls (Table A1). Of all garden-related activities, only growing leaf or root vegetables was more common among patients (OR = 2.5). The location of the garden showed no remarkable influence. Patients were also more likely to enter forests for vocational reasons than were controls (OR = 2.8) and were more likely to have collected wood (OR = 4.7).

Eating unwashed or uncooked vegetables, salads, herbs, berries, or mushrooms did not appear to be an important risk factor for alveolar echinococcosis; only eating unwashed strawberries or chewing grass was more common among patients than controls (OR = 2.2 and 4.4, respectively), and attributable risk calculations suggested these exposures could at most account for only a quarter of the overall risk for alveolar echinococcosis (Table A1). Drinking water from natural sources had no identifiable association with the disease.

In order to describe, simply, persons at risk among the study population, a specific risk score was derived from the 22 factors with p values <0.05; we chose only those factors with low interdependencies. Eight of the 22 factors were not strongly associated with any of the other variables (Table A2). The remaining variables with high interdependencies were selected as follows: living in a farmhouse was chosen instead of haymaking since including a three-level variable would have required weighing this factor; leaving the dog in the garden unattended was favored instead of six other dog-related factors (dog ownership, allowing the dog into the house, playing with the dog, walking the dog without leash, having a dog that ate mice, infrequent deworming of the dog) since it was a more relevant risk than dog ownership alone and was more reliably observed by the owners than the other factors. Cats left outdoors unattended was chosen as a risk factor instead of cats eating mice for the same reasons; being a farmer was chosen since it best represents the factors with which it was correlated (working in fields, pastures, grain fields).

Thus, the score was composed of 11 variables (Table A1): owning dogs that kill game, living in a farmhouse, owning dogs that roam outdoors unattended, collecting wood, being a farmer, chewing grass, living in a dwelling close to fields, going into forests for vocational reasons, growing leaf or root vegetables, owning cats that roam outdoors unattended, and eating unwashed strawberries. The score (range 0–11 points) was computed for 141 participants (37 patients, 104 controls); 19 participants had missing values in at least 1 exposure factor. The distribution among patients had a median of 6 score points (range 2–10); the distribution among controls had a median of 3 score points (range 0–9) (Figure). Of the patients, 81% had score values >4, but only 39% of the controls had score values >4.

**Figure:**
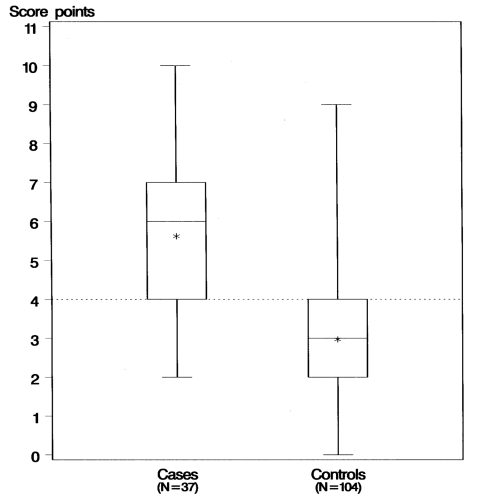
Risk score for alveolar echinococcosis in cases and controls. The plot presents minimum, 25th percentiles, median, 75th percentiles, and maximum values of the score points in cases and controls. The asterisk indicates the mean.

## Discussion

This study identified several possible important risk factors for acquiring alveolar echinococcosis. Farming was perhaps the most important risk factor identified; more than three quarters of patients were farmers, and attributable risk calculations suggested that almost two thirds of the cases could be accounted for by farming. The apparent risk with farming supports the view that substantial environmental contamination can be expected in open areas. The parasite's eggs can survive and remain infective for months under favorable conditions (high humidity, low temperatures) (*13*); thus, soil-related exposures are plausible. The finding that haymaking in meadows adjacent to streams or rivers bears a higher risk than haymaking in other areas agrees with the finding that more infected foxes are found close to water than in other habitats (*14*).

Although farming was an important risk factor, having a garden was not. An explanation may be that gardens usually cover a small area, and working in a garden requires less time, thus reducing exposure. Growing leaf or root vegetables was the only garden-related risk factor for A alveolar echinococcosis. The risk potential of growing specific garden produce may be interpreted in light of the greater amount of care and activity required for annual plants (leaf or root vegetables, salad vegetables), the fact that they are usually grown on larger patches than perennial herbs and strawberries, and the intense soil contact that occurs during harvesting.

Pet animals might pose a risk because of their close contact to humans and their contamination of soil around houses and in gardens. We found an association of dog ownership with acquisition of alveolar echinococcosis, and a lower but still relevant relationship with owning cats that roam freely outdoors or eat mice. The factor with the strongest association with the disease was "dogs that killed game," which is a rare disobedient behavior of individual dogs. Therefore, the attributable risk was lower than for the other variables related to dog ownership. Several other studies have indicated that dogs and cats are important risk factors for alveolar echinococcosis, although findings have been inconsistent (*9**,**10*). In China, an extensive inquiry with >2,500 participants including 86 patients with alveolar echinococcosis found that the number of dogs owned over time and the degree of dog contact were the most important risk factors (*15*).

Our results attach greater importance to ownership of dogs than of cats, particularly when the dog had activities possibly resulting in increased contact with soil or game. This finding is supported by experimental infection studies in which dogs proved to be susceptible to *E. multilocularis* eggs to the same high extent as foxes, and high worm loads developed; by contrast, cats had lower susceptibility and a slower maturation of the parasite (*6**,**16*). In the light of these findings, dogs and cats likely become a risk factor mainly by being infected themselves, in addition to transferring the eggs from fox feces or soil in their fur. Natural infections of pets have rarely been investigated systematically. The largest study on live cats and dogs from disease-endemic areas found coproantigen rates of 0.8% for both species (*17*).

In Austria, a strong association was found between hunting and the risk of acquiring alveolar echinococcosis (OR = 7.8) (*10*); however, a similar association was not shown in our study. Only 1 of 40 patients reported hunting. In Alaska, where hunting activities are more frequent than in Germany, no association between hunting and alveolar echinococcosis was observed (*9*). In China, no association was found between fox hunting and the disease (*15*).

Of all activities in the woods, only collecting wood was a likely important risk factor for alveolar echinococcosis, as indicated by the high OR and attributable risk calculations. Possibly, collecting wood posed a risk through contact with contaminated soil when a person picked the wood up from the ground, or the wood itself became contaminated when stacked in places accessible to roaming animals (clearings, forest perimeters, exterior parts of walls, open barns).

Chewing grass and eating unwashed strawberries were the only two variables of food consumption associated with alveolar echinococcosis. This risk may be attributable to ingestion of eggs from contaminated plant parts or from soil-contaminated hands. Other garden produce and mushrooms from fields and meadows were only rarely consumed raw and unwashed. Berries from the woods were more frequently consumed raw and unwashed than strawberries. The reasons why only strawberries constitute a risk include the fact that forest areas may be less likely to be egg-contaminated or that strawberries are eaten in larger quantities. The two case-control studies of Alaska and Austria found no association of alveolar echinococcosis with picking and eating raw produce from gardens, or berries and mushrooms from fields and forests (*9**,**10*).

This study had several important limitations. First, the long latent period for alveolar echinococcosis precluded determining the exact period relevant for an exposure. We restricted the assessment of most variables to the 10 years preceding the diagnosis of a case; we also restricted eligibility to diagnoses since 1990, which had the advantage that diagnoses were probably ascertained "early" after the patients' infection owing to improved diagnostic technology and greater awareness over time. The case-control studies on alveolar echinococcosis published previously included cases irrespective of diagnosis dates. Furthermore, in Austria, the observation period spanned the 20 years preceding diagnosis, and the study included data about deceased patients (*10*). In Alaska the time frame encompassed the whole lifetime of the participants (*9*). Second, many possible risk factors were correlated with each other, and eliminating possible confounding factors was not possible. In our analyses, we omitted multiple logistic regression because of the multicollinearity of the factors. In such a situation, variable selection procedures in multiple logistic regression might lead to the arbitrary removal of important factors from the final model. In our opinion, interpreting such a reduced risk model might be misleading, especially if recommendations for preventive measures were derived from these models alone. Instead, we considered different degrees of exposure between cases and controls. We constructed an unweighted risk score from high risk variables that were not strongly dependent on each other. Patients were more likely to have been exposed to a greater variety of potential risks during the defined exposure time, which speaks for a possible cumulative effect of potentially hazardous activities. Third, the matching of case-patients and controls by location could have selected for similar behavior among them, and thus falsely reduced the observed strength of associations of possible risk factors.

We conclude that farmers, compared to persons in other occupations, are at high risk for alveolar echinococcosis in endemic areas in Germany. The disease should be strongly suspected in farmers living in these areas who have symptoms suggestive of this disease. Since no single farming-related activity alone likely accounts for this risk, general measures to reduce possible exposure during farming (e.g., wearing gloves when handling soil, plants, or wood; washing hands before taking meals after farming) might best reduce this risk. The risk observed with haymaking suggests a need to evaluate a possible role of inhalation; although evidence is lacking, wearing protective masks in very dusty conditions during such work may minimize risk. Our data also suggest that dogs and cats may pose a risk and that an adequate anthelmintic prophylaxis (praziquantel at monthly intervals) may possibly reduce this risk. Finally, our data suggest that cleaning produce from fields or gardens may help to reduce the risk for this disease.

Until the early 1980s, human alveolar echinococcosis was known to occur in four countries of western and central Europe: Austria, France, Germany, and Switzerland (*5*). Since the 1990s, sporadic cases have been found in Belgium, Poland, and Greece (*8*); a first case report from Slovakia dates from 2000 (*18*). These cases suggest that the disease is spreading. Since eliminating the parasite is unfeasible, the population in the disease-endemic areas should be advised to adhere to personal cautionary measures to prevent new infections.

## Appendix

**Table A1:** Association between exposure factors and alveolar echinococcosis^a,b,c^

Risk factor	Patients		Controls		Crude			
	+	–	+	–	OR	95% CI	p value	AR (%)
Contact with definitive hosts								
Foxes								
Went hunting	1	39	5	111	0.6	0.1–5.1	0.641	
Shot and skinned foxes	1	39	1	115	–	–	–	
Saw a fox in the garden	8	32	14	102	2.0	0.7–5.4	0.181	
Saw a fox at the hen coop	8	28	23	76	1.0	0.4–2.3	0.941	
Dogs								
**Dog ownership (owned dogs or cared for others' dogs)**	**21**	**19**	**31**	**89**	**4.2**	**1.7–9.9**	**0.001**	**40.0**
**Allowed dog into the house**	**17**	**23**	**30**	**89**	**2.5**	**1.1–5.7**	**0.028**	**25.5**
Allowed dog into bedroom	3	37	5	113	1.8	0.4–7.5	0.421	
Was licked by dog	11	27	22	91	1.8	0.7–4.5	0.196	
**Played with the dog**	**16**	**24**	**29**	**90**	**2.6**	**1.1–6.2**	**0.037**	**24.6**
Brushed the dog's fur	13	27	24	95	2.3	0.9–5.5	0.076	
**Left dog in garden unattended** ^d^	**20**	**20**	**23**	**96**	**6.1**	**2.4–15.8**	**< 0.001**	**41.8**
**Walked dog without leash**	**15**	**25**	**18**	**101**	**4.2**	**1.7–10.8**	**0.003**	**28.6**
Walked dog without leash in forest	7	31	10	100	2.3	0.8–6.8	0.129	
**Dog killed game** ^d^	**6**	**34**	**1**	**117**	**18.0**	**2.2–149.5**	**0.008**	**14.2**
**Dog ate mice**	**7**	**22**	**7**	**77**	**3.1**	**1.0–9.5**	**0.043**	**16.4**
Dog had worms	3	35	4	104	2.1	0.5–9.5	0.326	
**Had dog dewormed infrequently or never**	**20**	**19**	**25**	**87**	**5.6**	**2.1–14.6**	**< 0.001**	**42.1**
Dog defecated in the house	3	36	13	102	0.7	0.2–2.5	0.540	
Cats								
Cat ownership (owned cats or cared for others' cats)	18	22	34	86	2.1	1.0–4.5	0.051	
Allowed cat into the house	14	26	31	89	1.6	0.7–3.4	0.262	
Allowed cat into the bed	3	37	17	103	0.5	0.1–1.8	0.276	
Was licked by the cat	4	36	20	98	0.5	0.2–1.7	0.307	
Fed the cat	10	30	26	93	1.2	0.5–2.8	0.683	
Brushed the cat's fur	2	37	8	107	0.7	0.1–3.9	0.684	
**Left cat unattended outdoors** ^d^	**18**	**22**	**33**	**87**	**2.3**	**1.0–4.8**	**0.038**	**25.4**
**Cat ate mice**	**16**	**22**	**27**	**84**	**2.3**	**1.0–5.2**	**0.038**	**23.8**
Had a cat toilet in the house	5	33	19	93	0.7	0.2–2.3	0.601	
Cleaned cat toilet	5	33	16	96	0.9	0.3–2.9	0.886	
Cat had worms	4	34	7	105	2.0	0.5–8.5	0.363	
Had cat dewormed infrequently or never	16	22	30	82	2.1	0.9–4.6	0.068	
Cat defecated in the house	6	31	18	91	1.0	0.3–2.8	0.929	
Contact with contaminated soil or dust								
Farming, living conditions								
**Was a farmer (fulltime or parttime)** ^d^	**33**	**7**	**65**	**55**	**4.7**	**1.8–12.1**	**0.001**	**64.9**
**Worked in pastures**	**30**	**8**	**52**	**59**	**5.7**	**2.1–15.7**	**< 0.001**	**65.1**
**Worked in fields**	**29**	**9**	**53**	**59**	**6.1**	**2.0–18.4**	**0.002**	**63.8**
**Worked in grain fields**	**25**	**12**	**48**	**59**	**3.0**	**1.3–7.3**	**0.013**	**45.0**
**Did haymaking**								
**In meadows not adjacent to water**	**10**	**16**	**14**	**95**	**5.9**	**1.9–18.8**		
**In meadows adjacent to water**	**14**	**16**	**11**	**95**	**12.9**	**3.6;46.2**	**< 0.001**	**52.9** ^e^
**Lived in a farmhouse** ^d^	**17**	**23**	**14**	**106**	**6.4**	**2.5–16.5**	**< 0.001**	**35.9**
Dwelling was adjacent to a forest	9	30	43	73	0.4	0.2–1.1	0.077	
Dwelling was adjacent to meadows	33	6	101	15	0.8	0.3–2.2	0.697	
**Dwelling was adjacent to fields** ^d^	**28**	**11**	**55**	**62**	**3.0**	**1.3–6.7**	**0.009**	**47.9**
Owned a hen coop	26	13	66	47	1.6	0.7–3.6	0.277	
Gardening								
Owned or had access to a garden	37	3	109	11	1.2	0.3–4.7	0.746	
Garden was close to a forest, meadows, or water	28	10	67	45	1.9	0.8–4.3	0.132	
Worked in the garden								
Frequently	20	9	74	22	0.6	0.2–1.7		
Occasionally	11	9	22	22	1.2	0.4–3.6	0.315	
Grew herbs	33	6	90	27	1.7	0.6–4.6	0.296	
Grew salad vegetables	32	8	78	42	2.4	1.0–6.1	0.060	
Grew strawberries	25	15	61	59	1.7	0.8–3.6	0.185	
**Grew root or leaf vegetables** ^d^	**32**	**8**	**77**	**43**	**2.5**	**1.0–6.4**	**0.049**	**48.0**
Did haymaking in the garden	28	12	84	35	1.0	0.4–2.2	0.972	
Raked up leaves in the garden	23	11	66	35	1.2	0.5–2.7	0.745	
Picked up windfalls	24	16	62	54	1.3	0.6–2.8	0.461	
Activities in forests								
Went into the forests	39	1	108	12	4.4	0.6–35.4	0.161	
**Went into forests for vocational reasons** ^d^	**9**	**31**	**11**	**109**	**2.8**	**1.1–7.4**	**0.036**	**14.5**
Went for a walk	28	9	91	18	0.6	0.3–1.6	0.316	
**Collected wood** ^d^	**24**	**16**	**28**	**90**	**4.7**	**2.1–10.4**	**< 0.001**	**47.2**
Picked mushrooms	7	31	34	78	0.5	0.2–1.3	0.146	
Went jogging	6	31	16	86	1.1	0.4–3.1	0.825	
Picked low growing berries	10	28	27	84	1.2	0.5–2.7	0.742	
Picked high growing berries	13	25	30	83	1.4	0.7–3.0	0.372	
Cleaned berries	23	14	60	38	1.0	0.5–2.3	0.921	
"Food" factors								
Ate unwashed herbs	6	34	15	104	1.2	0.4–3.5	0.681	
Ate unwashed lettuce	0	40	1	119	Undef.			
Ate unwashed vegetables	4	36	5	115	2.4	0.6–8.9	0.192	
**Ate unwashed strawberries** ^d^	**19**	**20**	**34**	**82**	**2.2**	**1.1–4.7**	**0.034**	**26.6**
Ate sorrel (*Rumex* sp.)	6	32	10	103	1.9	0.7–5.3	0.245	
**Chewed grass** ^d^	**14**	**25**	**14**	**101**	**4.4**	**1.7–11.2**	**0.002**	**27.7**
Ate raw berries	30	10	70	37	1.6	0.7–3.6	0.296	
Washed berries before eating	27	12	61	42	1.5	0.7–3.4	0.325	
Ate raw mushrooms	1	37	2	107	1.5	0.1–16.5	0.740	
Drank water from springs	11	29	23	91	1.4	0.6–3.2	0.391	

^a^Crude odds ratios (OR) based on simple conditional logistic regression analyses. ^b^CI, confidence intervals; AR, attributable risk. Factors with p < 0.05 are indicated in **boldface**. ^c^Exposure versus reference: +, number of persons exposed; –, number of pesons unexposed. ^d^Factors included in the risk score. ^e^AR was calculated from OR by using the exposure variable with two categories ("yes" and "no"; OR = 8.5; 24/40 cases were exposed).

**Table A2:** Association between exposure factors (Cramer's V)^a^

Variable	1	2	3	4	5	6	7	8	9	10	11	12	13	14	15	16	17	18	19	20	21	22
1 Owned dogs / cared for others' dogs	1.00	**0.94**	**0.91**	**0.89**	**0.74**	0.32	**0.66**	**1.00**	0.13	0.12	-0.05	-0.01	-0.04	-0.04	0.15	0.23	0.04	0.05	0.22	0.21	-0.10	0.07
2 Allowed the dog into the house		1.00	**0.88**	**0.82**	**0.69**	0.27	**0.62**	**0.95**	0.15	0.14	-0.06	-0.03	-0.05	-0.04	0.11	0.20	0.00	0.02	0.21	0.20	-0.14	0.07
3 Played with dog			1.00	**0.81**	**0.71**	0.21	**0.62**	**0.92**	0.14	0.13	-0.08	-0.05	-0.08	-0.08	0.08	0.15	0.03	0.00	0.27	0.19	-0.14	0.12
4 Left dog in garden unattended				1.00	**0.67**	0.36	**0.70**	**0.89**	0.22	0.21	-0.01	0.01	0.04	0.03	0.17	0.24	0.03	0.08	0.28	0.22	-0.08	0.13
5 Walked dog without leash					1.00	0.27	**0.59**	**0.78**	0.11	0.11	-0.01	0.03	0.00	0.01	0.11	0.10	0.01	-0.09	0.23	0.20	-0.03	0.08
6 Dog killed game						1.00	0.29	0.31	0.12	0.14	0.04	0.07	0.07	0.07	0.17	0.13	0.02	0.08	0.30	0.25	0.10	0.14
7 Dog ate mice							1.00	**0.66**	0.27	0.28	0.11	0.20	0.15	0.13	0.26	0.34	0.19	-0.04	0.35	0.43	0.00	0.09
8 Had dog dewormed infrequently or never								1.00	0.11	0.10	-0.04	-0.01	-0.04	-0.03	0.14	0.21	0.04	0.03	0.23	0.24	-0.07	0.10
9 Left cat outdoors unattended									1.00	**0.97**	0.19	0.23	0.26	0.21	0.37	0.41	0.08	0.15	0.19	0.24	0.09	0.07
10 Cat ate mice										1.00	0.22	0.26	0.30	0.24	0.38	0.38	0.10	0.15	0.22	0.18	0.11	0.08
11 Was a farmer											1.00	**0.88**	**0.87**	**0.83**	0.42	0.33	0.18	0.17	0.18	0.31	0.30	0.06
12 Worked in pastures												1.00	**0.88**	**0.83**	0.46	0.40	0.25	0.17	0.24	0.40	0.33	0.02
13 Worked in fields													1.00	**0.90**	0.43	0.37	0.24	0.22	0.24	0.32	0.36	0.09
14 Worked in grain fields														1.00	0.38	0.33	0.17	0.22	0.28	0.30	0.36	0.07
15 Did haymaking															1.00	**0.57**	0.21	0.25	0.40	0.37	0.20	0.13
16 Lived in a farmhouse																1.00	0.16	0.20	0.39	0.40	0.01	0.02
17 Dwelling was adjacent to fields																	1.00	0.15	0.06	0.20	-0.03	0.10
18 Grew root or leaf vegetables																		1.00	0.14	0.24	0.36	0.21
19 Went to forests for vocational reasons																			1.00	0.38	0.01	0.07
20 Collected wood																				1.00	0.24	0.10
21 Ate unwashed strawberries																					1.00	0.17
22 Chewed grass																						1.00

^a^Cells in bold mark Cramer's V > 0.5. High values mark strong associations.
